# Early diagnosis of thoracic spinal dural arteriovenous fistula using lumbar magnetic resonance imaging: A case report

**DOI:** 10.1002/ccr3.8309

**Published:** 2024-01-03

**Authors:** May Pyae Kyaw, Tatsuya Tanaka, Satoshi Anai, Yukinori Takase, Kiku Kamitoko, Hiromu Minagawa, Motohiro Yukitake, Junpei Sasaki, Oya Nagata, Akira Matsuno, Tadatsugu Morimoto

**Affiliations:** ^1^ Department of Neurosurgery Kouhoukai Takagi Hospital Okawa Japan; ^2^ Department of Neurosurgery International University of Health and Welfare Narita Hospital Narita Japan; ^3^ Department of Neurology Kouhoukai Takagi Hospital Okawa Japan; ^4^ Department of Orthopedic Surgery Kouhoukai Takagi Hospital Okawa Japan; ^5^ Department of Orthopedic Surgery, Faculty of Medicine Saga University Saga Japan

**Keywords:** gait disturbance, intermittent claudication, overlooked case, spinal dural arteriovenous fistula, thoracic vertebrae

## Abstract

In middle‐aged and older men, clinicians often suspect lumbar spine disease when gait is impaired with intermittent claudication, but spinal dural arteriovenous fistula (SDAVF) may be the etiology. An understanding of the key magnetic resonance imaging findings of SDAVF is necessary for early diagnosis, appropriate treatment, and minimization of complications.

## Graphical abstract

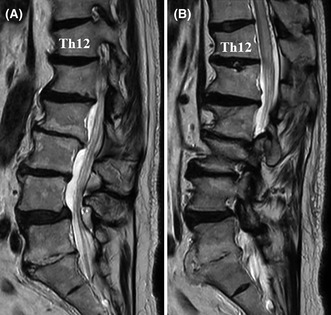

## 1 INTRODUCTION

Spinal dural arteriovenous fistula (SDAVF) is the most common spinal arteriovenous shunt disease and occurs mostly at the thoracic spine level in middle‐aged and older men. A fistula exists between the radiculomeningeal artery and the radiculomedullary vein in the dura mater near the spinal nerve root, and high pressure from the artery is applied to the veins around the spinal cord, causing the veins on the surface of the spinal cord to expand tortuously. This condition causes progressive myelopathy because of venous congestion and severe neurological symptoms. This disease progresses for several months to several years, resulting in irreversible spinal cord damage; however, if diagnosed early and the fistula is closed, disease progression can be halted, and recovery can be expected.^1^ However, this disease can be frequently confused with other medical conditions, and reaching a definitive diagnosis often takes a long time.^2^

Herein, we present the characteristic preoperative and postoperative images of a patient with SDAVF treated with endovascular embolization. This case report aims to address the diagnostic issues of this disease and emphasizes the need for early diagnosis using characteristic imaging features and treatment, which are critical in improving outcomes.

## 2 CASE REPORT

### 2.1 Case history and examination

A 77‐year‐old man with a history of lumbar spondylosis presented to our hospital complaining of progressive weakness and paresthesia in both lower limbs with gait impairment for 2 months. The patient had no history of spinal surgery or family history of any spinal disease. Neurological examination revealed no weakness (5/5) and decreased sensation (4/5) in the lower extremities and no vesicorectal involvement. Therefore, we suspected intermittent claudication due to lumbar spine disease.

**FIGURE 1:**
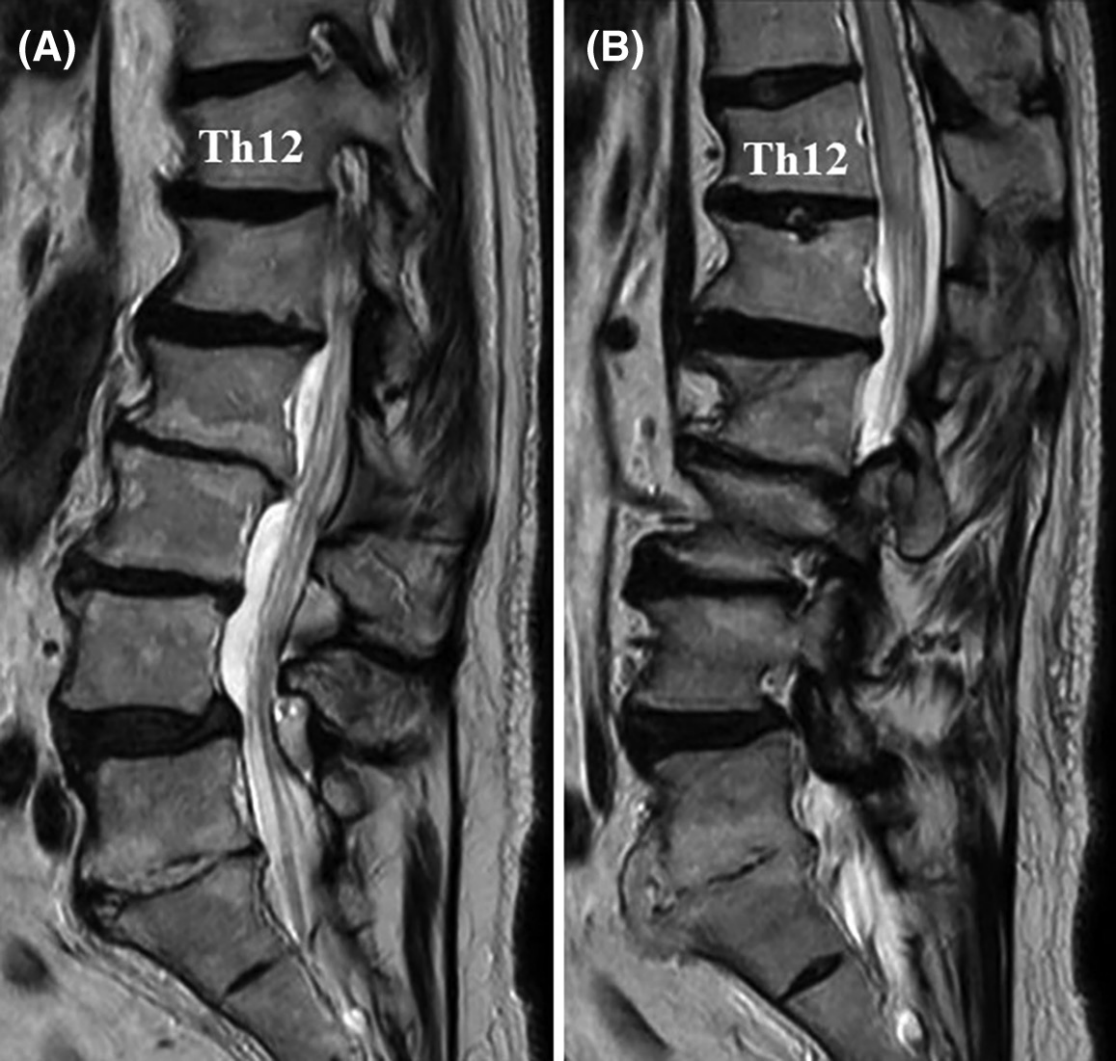
Initial magnetic resonance imaging (MRI) of the lumbar spine.(A, B) Sagittal T2‐weighted MRI showing lumbar spondylosis, spinal cord edema, and flow voids around the cord at the Th11–L1 level.

Initial evaluation with unenhanced magnetic resonance imaging (MRI) of the lumbar spine revealed abnormal thoracic spinal cord expansion in the T11–12 regions, with increased fluid signal within the spinal cord (Figure 1).

**FIGURE 2:**
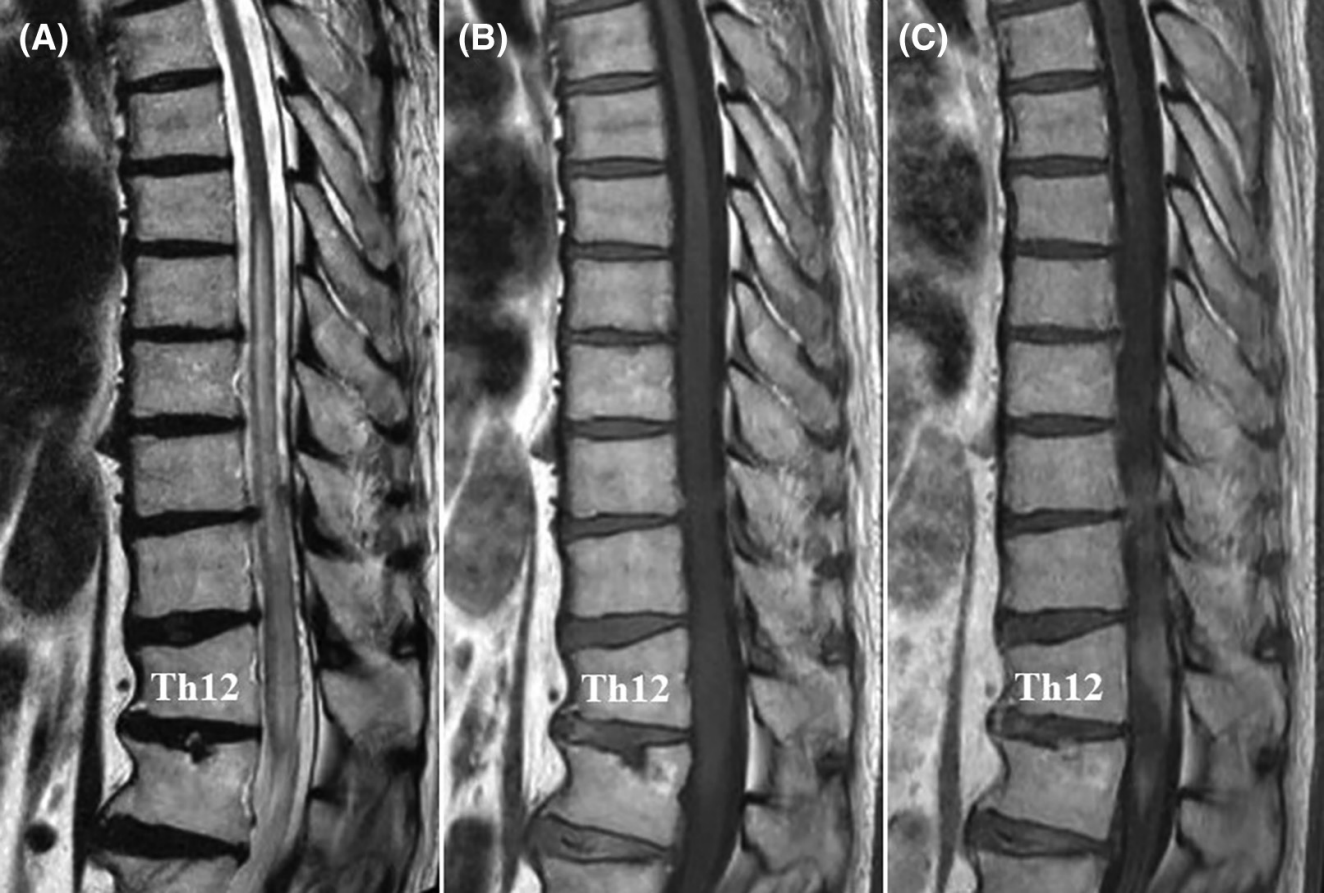
Preoperative thoracic magnetic resonance imaging (MRI). (A) Sagittal T2‐weighted MRI showing spinal cord edema and flow voids around the cord at the Th7–L1 level. (B) Sagittal T1‐weighted MRI showing spinal cord edema. (C) Sagittal contrast‐enhanced MRI showing heterogeneous enhancement of the spinal cord at the Th10–L1 level.

Magnetic resonance imaging of the thoracic spine showed abnormal thoracic spinal cord expansion in the T7–L1 regions, with increased fluid signal within the cord and numerous abnormally dilated vascular structures around the thoracic spine, consistent with SDAVF (Figure 2).

**FIGURE 3:**
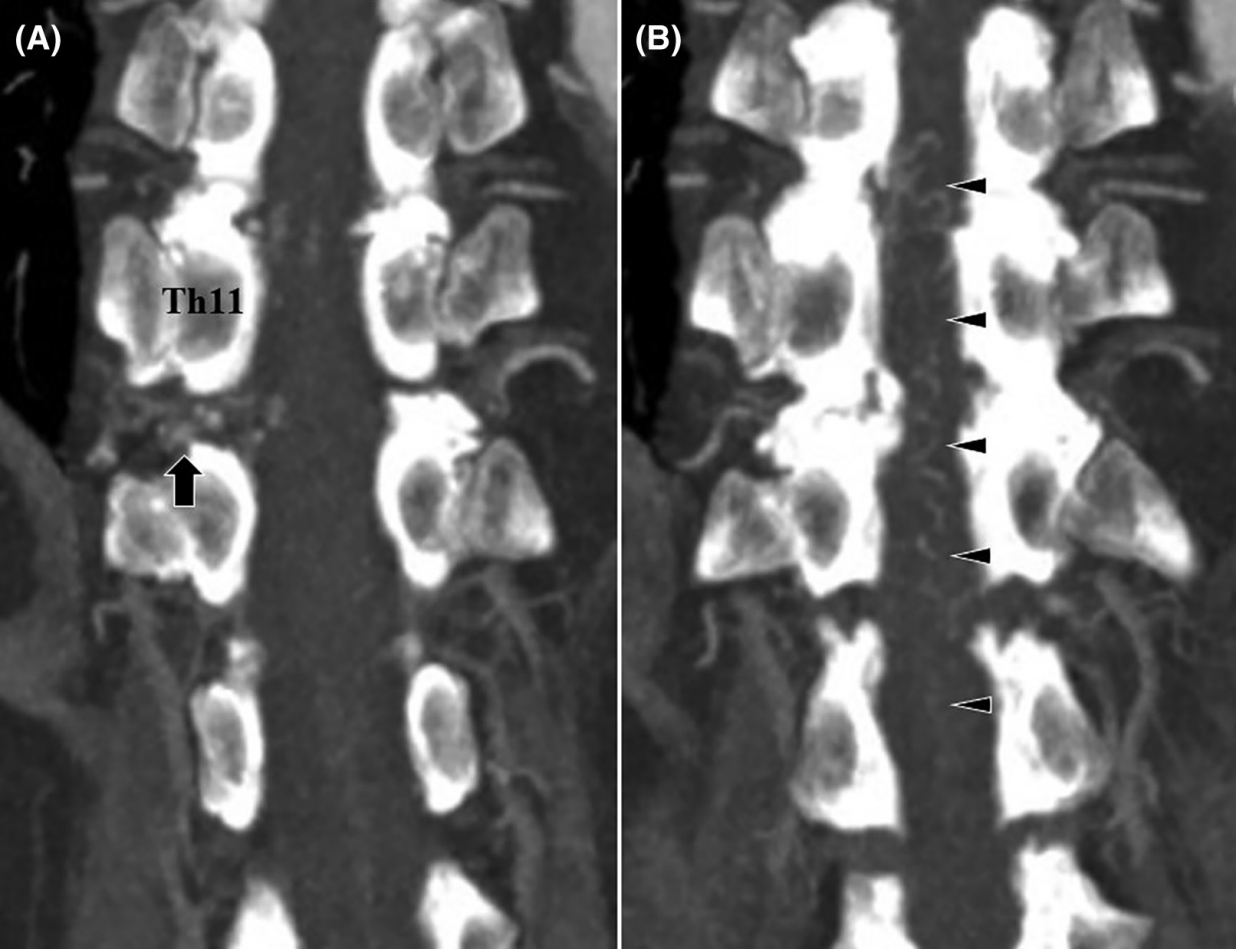
Preoperative computed tomography (CT) scan. (A, B) Coronal CT showing a premedullary vessel at the level of Th11/12 and a dilated premedullary drainage vein.

A multiplanar reconstruction (MPR) image from multidetector row computed tomography angiography (MDCTA) showed vessel entanglement below the right Th11 pedicle (Figure 3A). These vessels were connected to intraspinal tortuous vessels, suggesting perimedullary draining veins (Figure 3B).

**FIGURE 4:**
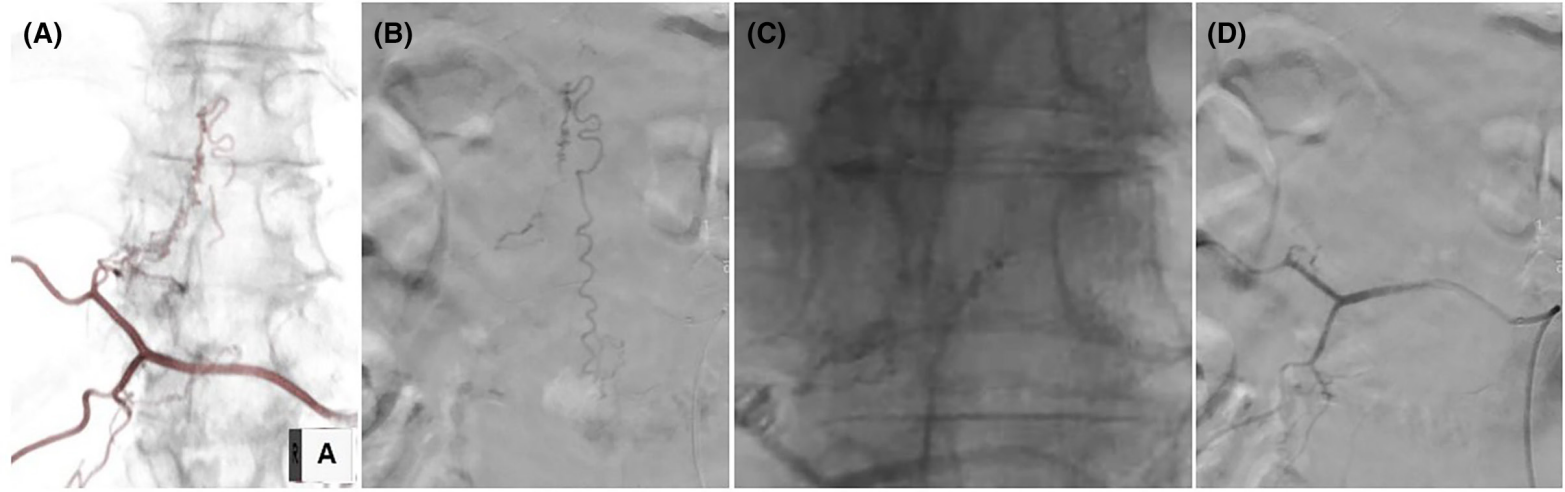
(A) Three‐dimensional digital subtraction angiography (DSA) image of the right Th11 and Th12 segmental arteries showing the spinal dural arteriovenous fistula (SDAVF), intradural arterialized vein, and dilated premedullary veins. (B) Anteroposterior (AP) view DSA image of the right Th11 segmental artery showing a shunt lesion from selective catheterization of the right Th11 radicular branch with a tangle of arterialized veins noted in the central spinal canal. The fistula is noted at the right Th11 nerve sheath. (C) Post‐treatment radiograph showing the presence of n‐butyl‐2‐cyanoacrylate. (D) Post‐treatment AP view DSA image of the right Th11 and Th12 segmental arteries showing the disappearance of SDAVF.

The patient underwent spinal angiography under local anesthesia. A left inguinal common femoral artery puncture was performed for vascular access using a 4‐French sheath, and selective spinal angiography was initiated using a 4‐French Shepherd hook catheter (Figure 4A). Specific and significant findings on angiography included a fistula supplied by the right Th11 radicular artery draining into the right Th11 radicular veins.

Angiography of the right Th11 radicular artery did not show the radiculomedullary and radiculopial arteries. These findings were discussed with neurologists, neurosurgeons, and an orthopedic surgeon, and it was initially decided to treat with embolization; however, if a complete cure could not be achieved, surgical treatment would be opted for.

### 2.2 Treatment

The patient was placed under general anesthesia to undergo superselective angiography and endovascular embolization. A right groin common femoral artery puncture was performed for vascular access using a 5‐French sheath. Superselective angiography and endovascular embolization with n‐butyl cyanoacrylate of the Th11 radicular artery were performed. Complete angiographic obliteration of the fistula was achieved (Figure 4).

### 2.3 Outcome and follow‐up

**FIGURE 5:**
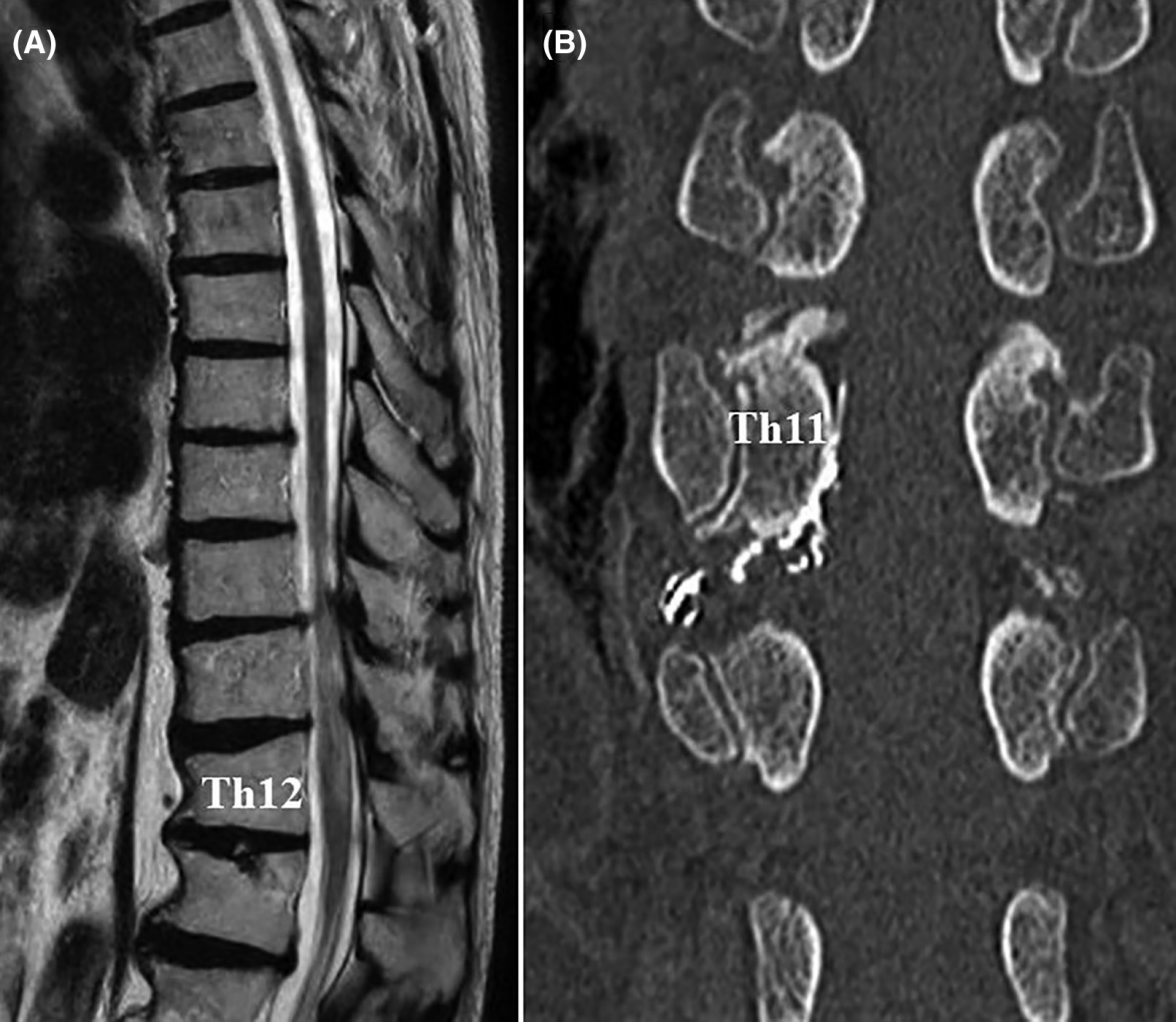
(A) Post‐treatment sagittal T2‐weighted MRI of the thoracolumbar spine showing the disappearance of edema and flow voids. (B) Coronal computed tomography revealing n‐butyl‐2‐cyanoacrylate at Th11/12 vertebral foramen.

The patient also experienced immediate improvement in sensory symptoms, which progressed with each postoperative day of recovery. The patient was discharged from the hospital 2 weeks after the treatment. Postoperative MRI revealed the expected improvement in spinal cord edema and the disappearance of abnormally dilated vascular flow voids around the spinal cord (Figure 5A), and coronal computed tomography revealed n‐butyl‐2‐cyanoacrylate at Th11/12 vertebral foramen (Figure 5B).

## 3 DISCUSSION

### 3.1 Neurological symptoms of SDAVF

A typical patient with SDAVF has symptoms associated with thoracic myelopathy, epiconus syndrome, and conus medullaris syndrome.^1^ Thoracic myelopathy includes motor weakness of the proximal lower extremities, posterior funiculus dysfunction, and exaggerated deep tendon reflexes. Epiconus syndrome encompasses a gradual onset of numbness that initiates in the distal regions of the lower limbs and progresses upward. Conus medullaris syndrome includes bowel and bladder dysfunction. Claudication has been associated not only with walking but also with bathing, drinking, sleeping, gardening, and even singing.^1^, ^3^ These symptoms are specific to SDAVF and differ significantly from those of spinal stenosis.^1^, ^3^ If symptoms cannot be explained by common spinal stenosis, the possibility of SDAVF should be considered.

### 3.2 Characteristic imaging findings of SDAVF

The most sensitive MRI finding in SDAVF is venous congestion of the spinal cord, with reported sensitivity rates of up to 100%.^4^ Most patients with SDAVF show high signal intensity with peripheral low signal intensity of the spinal cord on T2‐weighted MRI.^5^ Venous congestion is distributed from the lower thoracic spinal cord to the conus medullaris, regardless of the level of the fistula.^6^ Even if the fistula is located at the thoracic level, lumbar MRI may show venous congestion of the conus medullaris.

Dilated spinal cord veins in the subarachnoid space are a very specific finding in patients with SDAVF. The specificity of high signal intensity on T2‐weighted MRI and flow voids is 97%.^4^, ^7^ Although venous congestion may mimic intramedullary tumors, myelitis, syringomyelia, or demyelinating diseases of the spine, the presence of dilated veins may differentiate SDAVF from other conditions that mimic SDAVF.^8^, ^9^ T2‐weighted MRI shows these abnormal vessels as flow voids around the spinal cord. In some patients with SDAVF, MRI does not show flow voids. However, MDCTA and contrast‐enhanced magnetic resonance angiography (MRA) with gadolinium can help identify abnormal vessels around the spinal cord.^10^, ^11^ Even lumbar MRI can identify venous congestion and flow voids at the conus medullaris.^12^ If conus medullaris lesions are evident on a lumbar MRI, a thoracic MRI should be performed to verify the presence of venous congestion around the spinal cord and assess vascular flow voids near the cord.

### 3.3 Early diagnosis procedure for SDAVF

The reason for the delayed diagnosis of SDAVF is that it is easily misdiagnosed as another disease. Spinal degenerative diseases and myelitis are particularly common misdiagnoses.

The initial symptoms of SDAVF are nonspecific motor and sensory deficits in the lower extremities. Symptoms worsen with walking and improve with rest. These symptoms are similar to those of intermittent cauda equina claudication caused by lumbar spinal stenosis, which is a common disease. The first investigation is often a lumbar MRI scan. If spinal stenosis is suggested, it may be misdiagnosed as the cause. To avoid this misdiagnosis, considering whether the symptoms can actually be explained by lumbar spinal canal is important.

Spinal dural arteriovenous fistula is worsened not only by walking but also by singing, the Valsalva maneuver, and alcohol consumption.^1^, ^3^ Additionally, it is crucial to remain vigilant for symptoms that may be challenging to account for due to lumbar spinal lesions, such as weakness in the iliopsoas muscle, sensory disturbances extending beyond the groin, and the presence of the Babinski sign. Furthermore, SDAVF should be considered if symptoms are slowly worsening. Attention should be paid to the presence or absence of abnormalities in the thoracolumbar junction (intramedullary high signal intensity, abnormal vascular images in the subarachnoid space) on lumbar spine MRI.

Spinal dural arteriovenous fistula is easily misdiagnosed as myelitis because long intramedullary hyperintensities are observed on T2‐weighted images. When a long intramedullary hyperintensity is observed, one should be aware of the abnormal vascular image in the subarachnoid space that is characteristic of SDAVF. The course of SDAVF is often progress slowly; however, it can worsen rapidly with exercise. SDAVF is often misdiagnosed as myelitis, which has an acute onset.^13^ SDAVF often has a mild increase in the number of cells in the cerebrospinal fluid. The administration of steroids has been documented to lead to sudden clinical deterioration in individuals with SDAVF. When encountering a patient with myelitis whose symptoms worsen with steroid administration, SDAVF must be differentiated.^7^, ^14^

Spinal dural arteriovenous fistula is suspected based on clinical and spinal MRI findings. Next, minimally invasive three‐dimensional computed tomography, angiography, or contrast‐enhanced MRA is used to detect abnormal blood vessels. Subsequently, selective spinal angiography is employed to establish a conclusive diagnosis of SDAVF.

### 3.4 Treatment and outcomes of SDAVF

Spinal dural arteriovenous fistula treatment includes direct microsurgery and endovascular embolization. Clinical outcomes were determined by shorter preoperative symptom duration, the severity of initial deficit, the extent of spinal cord edema, treatment failure, and residual fistula.^8^ In particular, shorter preoperative symptom durations were significantly associated with improved motor function (median, 0.8 vs. 3.1 years; *p* = 0.001) and improved urinary function (median, 0.8 vs. 2.2 years; *p* = 0.040) postoperatively.^15^ Early diagnosis and treatment are important.

### 3.5 Reminder to clinicians

Spinal dural arteriovenous fistula is characterized by thoracic myelopathy. SDAVF is generally thought to be characterized by an early onset of paresthesia and intermittent lameness, followed by a slow progression of myelopathy. However, it is easily misdiagnosed as another disease such as spinal degenerative disease and myelitis.

However, SDAVF is a treatable condition, and early diagnosis and treatment can influence prognosis. Therefore, clinicians should aggressively and promptly evaluate suspected SDAVF. This is achieved by identifying lesions at the thoracic level through a comprehensive clinical assessment and recognizing the distinctive imaging features, which aid in making an accurate diagnosis and determining the suitable treatment plan.

## 4 CONCLUSION

Spinal dural arteriovenous fistula is an uncommon condition primarily impacting the spinal cord, characterized by venous congestion of the spinal cord stemming from a dural arteriovenous fistula. Although it may present with symptoms of intermittent lameness, it may not have typical symptoms because of its gradual progression. As in this study's case, this condition is often misdiagnosed as a lumbar spine disease, such as lumbar degenerative disease. Imaging plays a crucial role in achieving a precise diagnosis, with T2‐weighted images proving particularly valuable for the diagnosis of SDAVF. MRI sagittal sections show high signal intensity of the spinal cord and dilated spinal veins in the subarachnoid space. The treatment options encompass both direct microsurgery and endovascular embolization. Because SDAVF is treatable, early detection using diagnostic imaging is important.

## AUTHOR CONTRIBUTIONS

**May Pyae Kyaw:** Data curation; investigation; writing – original draft; writing – review and editing. **Tatsuya Tanaka:** Data curation; formal analysis; investigation; project administration; writing – original draft; writing – review and editing. **Satoshi Anai:** Data curation; investigation; writing – review and editing. **Yukinori Takase:** Data curation; investigation; writing – review and editing. **Kiku Kamitoko:** Data curation; investigation; writing – review and editing. **Hiromu Minagawa:** Data curation; investigation; writing – review and editing. **Motohiro Yukitake:** Data curation; investigation; writing – review and editing. **Junpei Sasaki:** Data curation; investigation; writing – review and editing. **Oya Nagata:** Data curation; investigation; writing – review and editing. **Akira Matsuno:** Supervision; writing – review and editing. **Tadatsugu Morimoto:** Supervision; writing – review and editing.

## FUNDING INFORMATION

None.

## CONFLICT OF INTEREST STATEMENT

The authors declare that they have no competing interests.

## ETHICS STATEMENT

Not mandated for case reports.

## CONSENT

Written informed consent to publish this report was obtained from the patient the journal's patient consent policy.

## Data Availability

Data pertaining to this case can be obtained by contacting the corresponding author.
